# Supplementary material for: A second genetic screen for gurken mRNA mislocalisation uncovers novel phenotypes of piRNA pathway mutants in Drosophila
Source: Biol Open. 2025 Nov 25;14(11):bio062321. doi: 10.1242/bio.062321 (PMC12690538; doi:10.1242/bio.062321)
Supplement: Supplementary information [file biolopen-14-062321-s1.pdf]

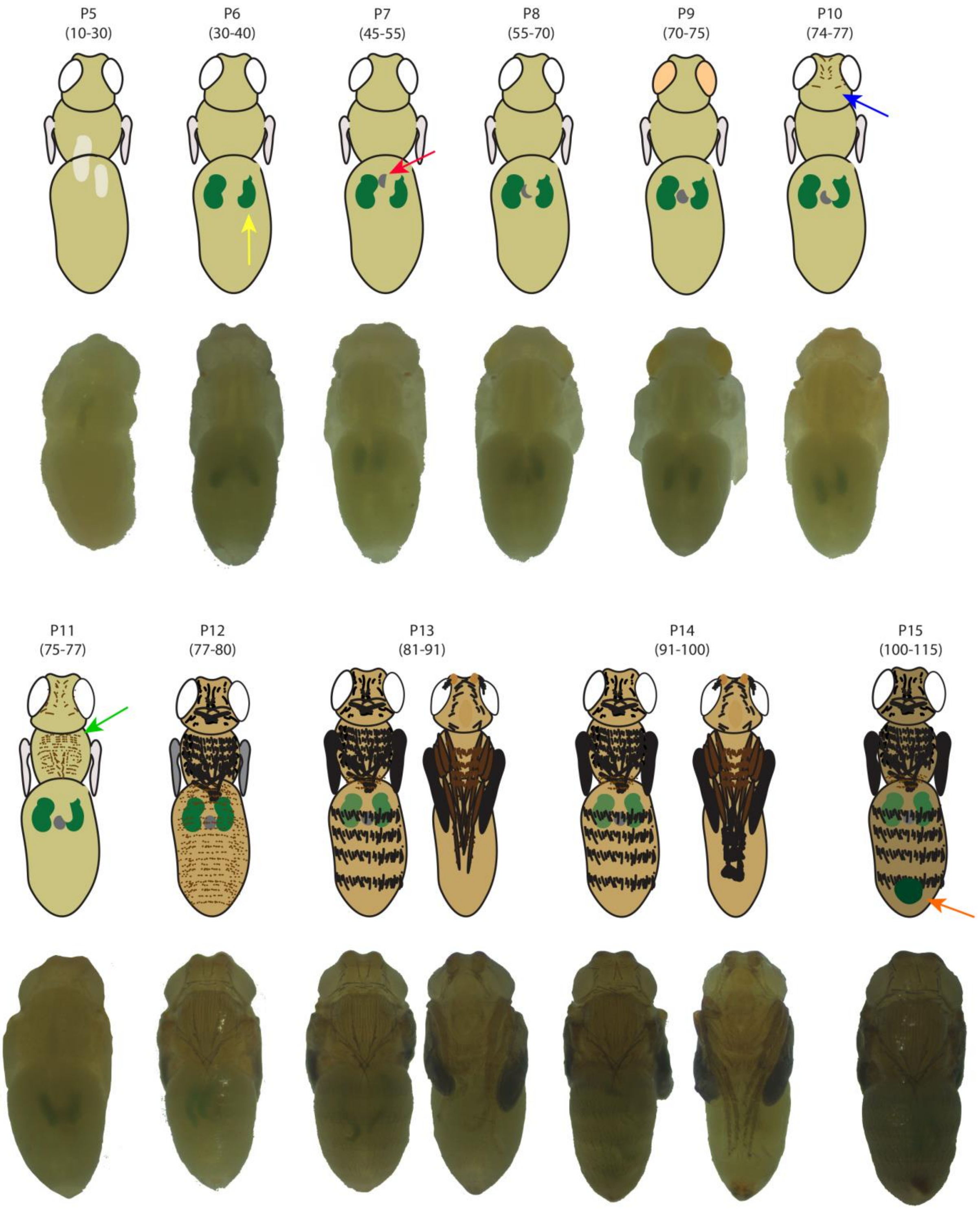

Fig. S1. Stages of pupal metamorphosis in *Drosophila melanogaster*.

Staging process adapted from Bainbridge and Bownes (1981) [37]. The entire pupal metamorphosis consists of 15 stages, of which only stages P5 and onwards are shown. w^{1118} flies are represented. Stages P5-P8 are dependent upon the presence or absence of the green Malpighian tubules (yellow arrow) and the relative position

Biology Open • Supplementary information

of the ‘yellow body’ (red arrow), which moves posteriorly between P7 and P8. P9 is characterised by an amber eye colour. As this is not present in w^{1118} flies, an alternative wild-type fly was used for this stage. P10 and P11 are determined by different levels of bristle formation on the head (blue arrow) and thorax (green arrow) of the fly. P12 begins with tanning of the pupa, as well as abdominal tergite formation and greying of the wings. P13 has black wings, fully developed tergites, and bristles on the legs. P14 differentiates from P13 as the P14 fly has visible, black claws. Finally, P15 is characterised by a visible meconium (waste build-up in the gut due to larval food intake, orange arrow). Numbers in brackets represent approximated developmental timings, represented as hours after puparium formation.

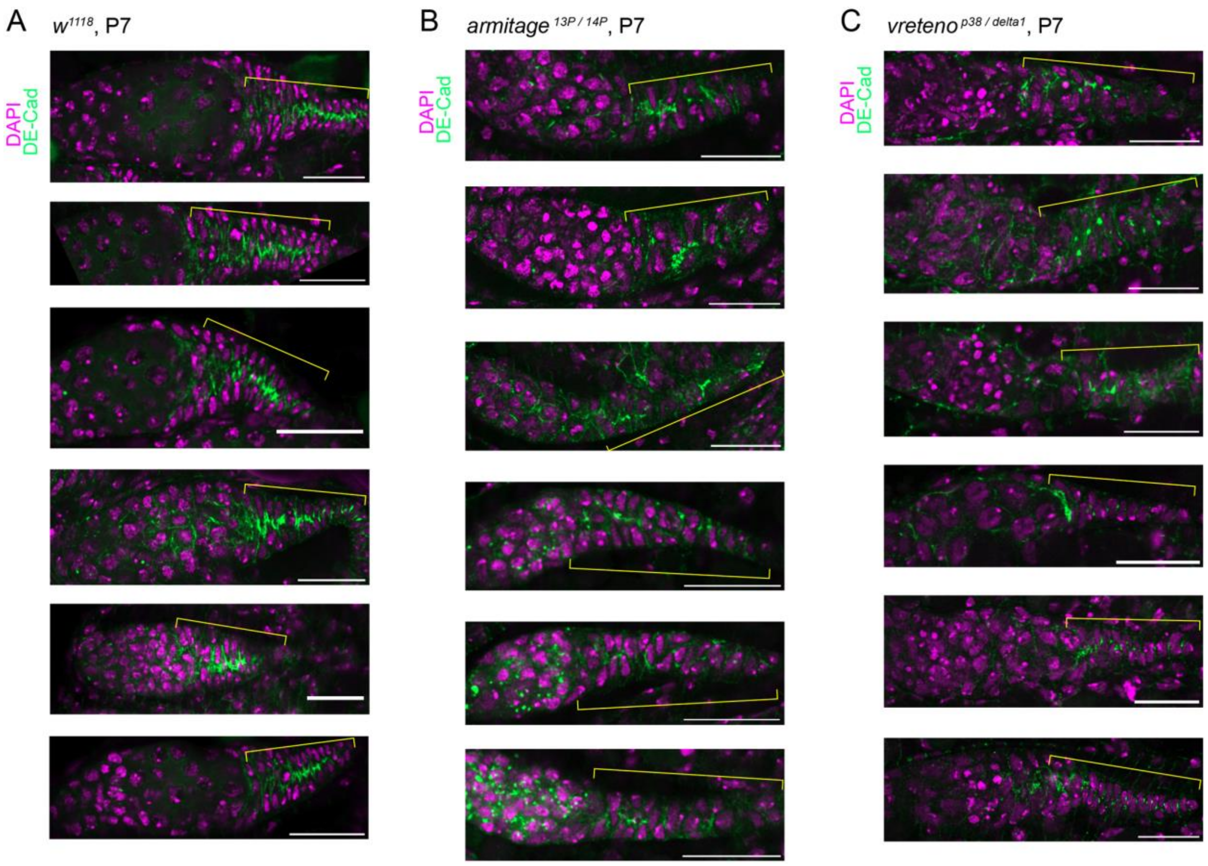

Fig. S2. Defective basal stalk formation in the piRNA pathway mutant pupal ovaries.

Shown are stage P7 ovarioles of indicated genotypes stained for DE-Cadherin. Images of six different ovarioles from each genotype are shown. The strong continuous signal of DE-Cadherin between intercalating basal stalk precursor cells (marked by yellow brackets) are consistently seen in the wild-type ovarioles, while the signals of DE-Cadherin are not continuous in the *armitage* and *vreteno* mutant ovarioles. Scale bars = 20 mm.

Biology Open • Supplementary information

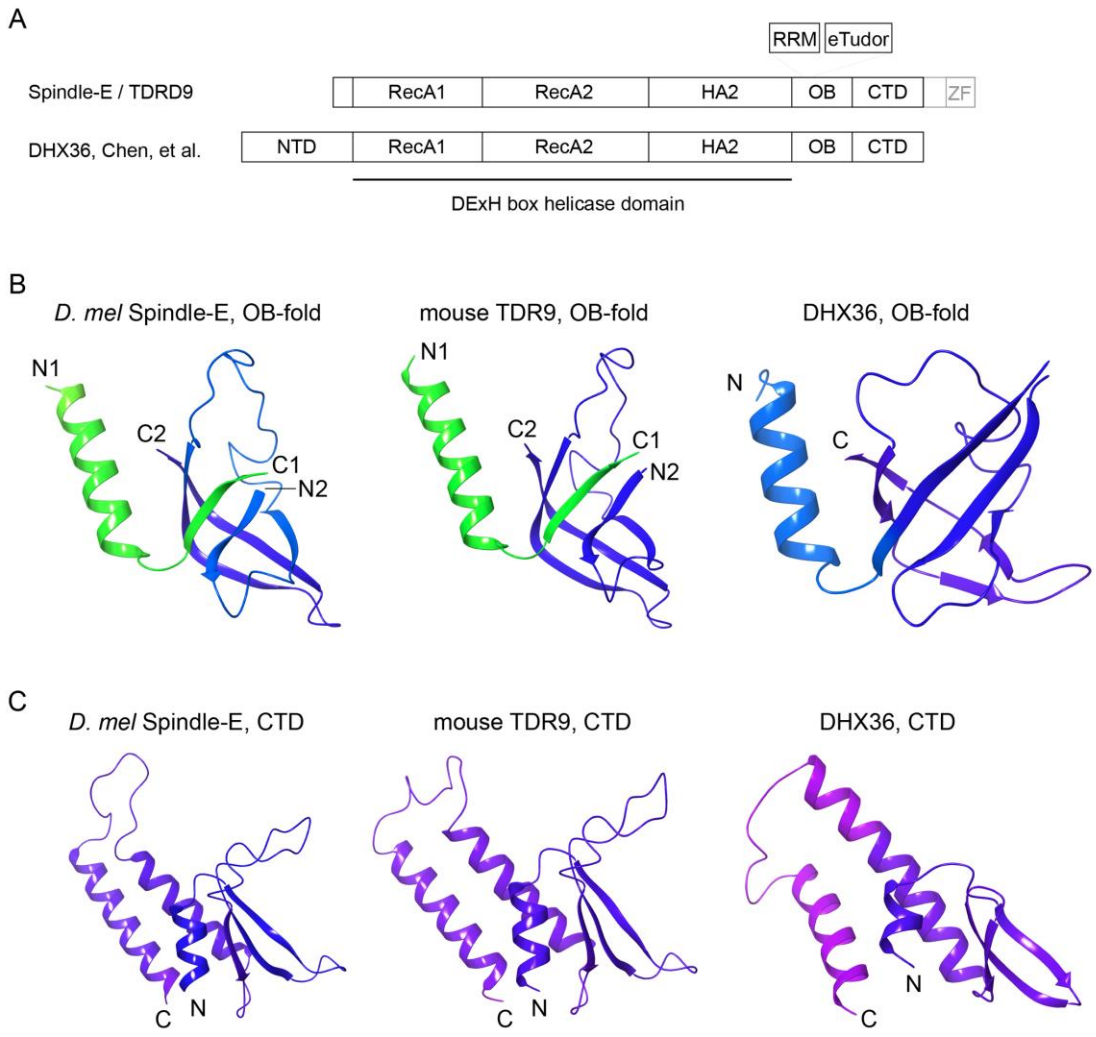

Fig. S3. Structural similarities between Spindle-E and DExH box RNA helicase-containing protein DHX36.

A. Domain architecture of Spindle-E/TDRD9 and Drosophila DHX36. The DExH box RNA helicase domain of DHX36 consists of two Rec A domains and helicase-associated 2 (HA2) domain [43]. DHX36 additionally has the N- and C- terminus domains (NTD and CTD) and an OB-fold. Spindle-E and TDRD9 carry the DExH box helicase domain, OB-fold and the CTD. RRM and extended Tudor (eTudor) domains are inserted in the middle of the predicted OB-fold. **B and C.** Alphafold-predicted structures of the OB-fold (in B) and the CTD (in C) of Spindle-E and TDRD9, showing similarities to the corresponding domains of DHX36 [43]. The predicted OB-fold of Spindle-E and TDRD9 is separated into two halves by RRM and eTudor domains but form intact domains in the whole protein structure. Regions shown for the structure are *D. mel* Spindle-E OB-fold: 714-738 + 1154-1212, mouse TDRD9 OB-fold: 714-739 + 1142-1201, *D. mel* Spindle-E CTD: 1217-1323, and mouse TDRD9 CTD: 1206-1312.

Biology Open • Supplementary information

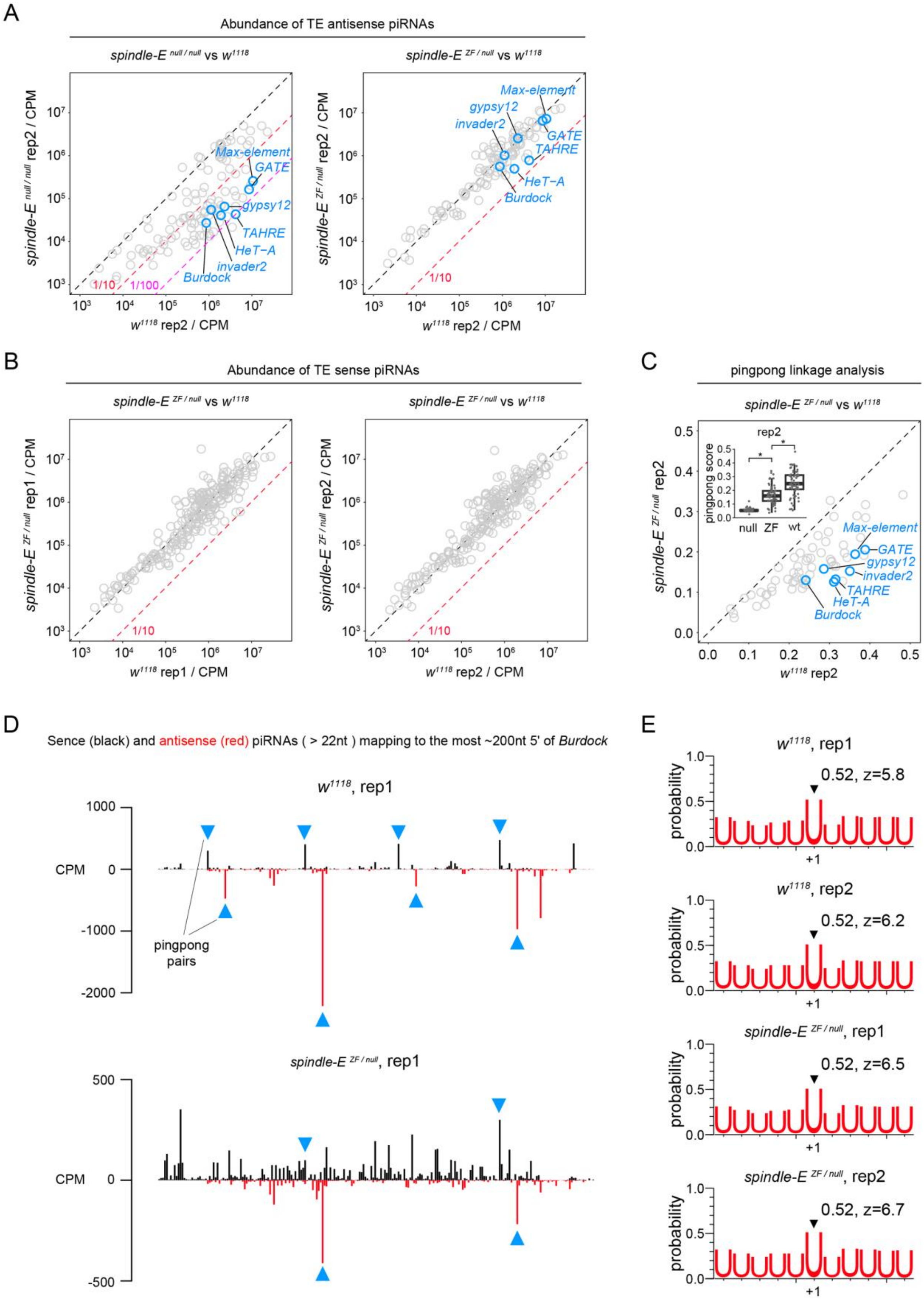

Biology Open • Supplementary information

Fig. S4. The Zinc Finger motif of Spindle-E is required for efficient ping-pong but not for phasing piRNA biogenesis.

A and B. Scatter plots showing the abundance of piRNAs mapping to antisense (in A) or sense (in B) transposon sequences. The plots compare the replicate 2 of the wild-type (w^{1118}), the Zinc Finger mutant (ZF) and the null mutants of *spindle-E* in A and replicates 1 and 2 of the wild-type (w^{1118}) and the Zinc Finger mutant in B. The same 7 representative transposons as Figure 6 are highlighted. **C.** Scatter plot comparing the ping-pong linkage values of transposon mapping piRNAs between the wild-type and the *spindle-E* Zinc Finger mutant. The replicate 2 is shown. *: student t test $p < 1.0 \times 10^{-15}$ **D.** Shown are the coverage of piRNA 5' ends mapping to the sense (black) and the antisense (red) strands of the 5' end region of *Burdock*. Arrowheads indicate ping-pong pairs, which are dominant in the wild-type but not in the *spindle-E* Zinc Finger mutant. **E.** Shown are frequencies of Uridine nucleotides found at the 11 nucleotides' window around the piRNA 3' ends that mapped to the antisense transposon sequences. "+1" position corresponds to the immediate downstream nucleotide of the piRNA 3' end.

Biology Open • Supplementary information

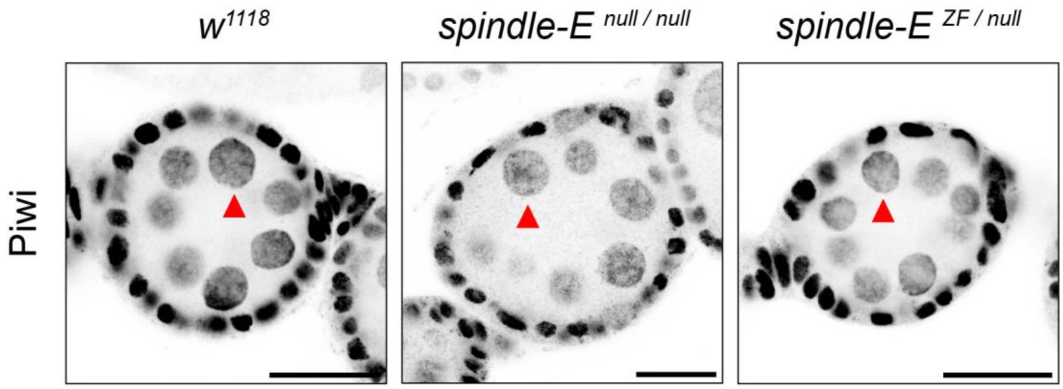

Fig. S5. Piwi localisation in the *spindle-E* mutants.

Shown are early-stage egg chambers of indicated genotypes stained for Piwi. The nuclear localisation of Piwi, marked by arrowheads, is affected neither in the *spindle-E* null mutant nor in the *spindle-E* Zinc Finger mutant egg chambers. Scale bars = 20 μm.

Biology Open • Supplementary information

Table S1. Sequences of short oligo DNA for the RNA fluorescent *in situ* hybridization.

Available for download at
https://journals.biologists.com/bio/article-lookup/doi/10.1242/bio.062321#supplementary-data

Table S2. Processed data of sequencing libraries that are used in figures. Tab 1: abundance of transposon sense and antisense piRNAs normalised to one million endogenous siRNAs. Tab 2: ping-pong linkage values of 63 representative Drosophila transposons. Tab 3: nucleotide frequencies around piRNA 3' ends, used as input for weblogo. Tab 4: normalised gene counts of RNA sequencing libraries after DESeq2. Tabs 5 and 6: results of the differential gene expression analysis after DESeq2.

Available for download at
https://journals.biologists.com/bio/article-lookup/doi/10.1242/bio.062321#supplementary-data
